# Cyclic alternating patterns and arousals: what is relevant in obstructive sleep apnea? In Memoriam Mario Giovanni Terzano

**DOI:** 10.1097/MCP.0000000000000825

**Published:** 2021-09-07

**Authors:** Liborio Parrino, Francesco Rausa, Nicoletta Azzi, Irene Pollara, Carlotta Mutti

**Affiliations:** Sleep Disorders Center, Department of General and Specialized Medicine, University Hospital of Parma, Parma, Italy

**Keywords:** cyclic alternating pattern, continuous positive airway pressure, excessive daytime sleepiness, obstructive sleep apnea

## Abstract

To review main knowledges and gaps in the field of sleep microstructure, represented by the cyclic alternating pattern (CAP), in obstructive sleep apnea (OSA).

**Recent findings:**

The (electroencephalographic and autonomic) ‘intensity’ of arousals in OSA patients, measured through the metrics of CAP, correlate with OSA severity and with disease burden. Continuous positive airway pressure determines variations in sleep architecture (conventional parameters) and at the microstructural level, at different time points.

**Summary:**

CAP is not only an ‘attractor’ of arousals, but also organizes distribution of K-complexes and delta bursts in non-rapid eye movement sleep. Although attention is always concentrated on the A-phase of CAP, a crucial role is play by the phase B, which reflects a period of transient inhibition. Respiratory events in OSA are a typical example of phase B-associated condition, as they occur during the interval between successive A-phases. Accordingly sleep microstructure provides useful insights in the pathophysiology and estimation of OSA severity and may be exploited to follow-up treatment efficacy. In the complex relationship among sleep fragmentation, excessive daytime sleepiness, cognition and cardiovascular risk the CAP framework can offer an integrative perspective in a multidisciplinary scenario.

## INTRODUCTION: KEEPING TIME IN BIOLOGICAL SYSTEMS

**FIGURE 1:**
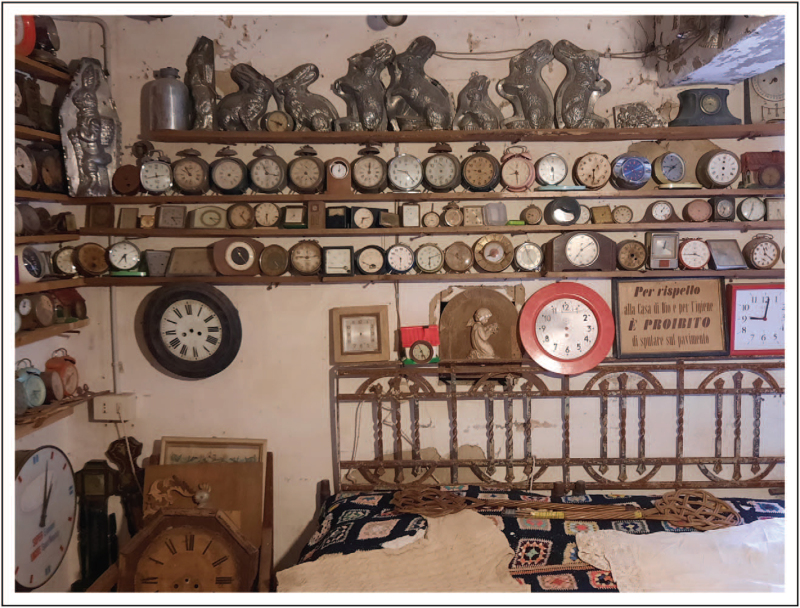
Bedroom hosting multiple types of alarm-clocks, ‘Ettore Guatelli Museum’, Parma, Italy.

Observing natural events, one realizes the existence of rhythmic phenomena that cover time spans of different lengths. They range from ultrashort rhythms (an electron oscillates 1.6 × 10^21^ radians per second) to phenomena that last hours such as the daily tidal rhythms or the opening of flowers, up to events lasting months or years, as in the case of hibernation, or prey/predator balances. There are also other rhythms hidden from direct observation, such as brain waves, hormone secretion and the activity of genes at the level of individual cells. But how does a biological system ‘keep time’? Some phenomena are linked to the movements of our planet, as in the case of sleep/wake rhythms, bounded to the alternation of day and night, or the times of hibernation linked to the different seasons. In mammals, the master clock is located in the suprachiasmatic nucleus and regulates the harmonic alternation of sleep and wake cycles over a near-24-h period and continuously modulated by environmental cues (Fig. 1) and genetic predisposition [1]. Sleep is a periodic phenomenon, which occurs at intervals of about 16 h. Most people rise at 7 a.m. and go to bed at 11 p.m., dedicating 1/3 of their daily life to sleep. Circadian and homeostatic processes modulate the duration and intensity of the sleep process. In physiological conditions, a powerful software determines the length and distribution of rapid eye movement (REM) and non-REM (N1, N2, N3) sleep stages. Their temporal organization across the night delineates the macrostructure of sleep. A series of macrostructural parameters provide information on the timing and continuity of sleep composition (Table 1). 

**Table 1:** Main features of nocturnal polysomnography

Sleep macrostructure					
Humans (except newborns) fall asleep in NREM sleep	Brain takes about 25 min to reach deep sleep (N3)	First episode of REM sleep appears about 10 min after the end of the first N3 period	N3 sleep prevails in the first part of the night, while stages N2 and REM dominate in the second half	REM sleep accounts for 20–25% of total sleep, deep sleep makes up 20–25%, while light sleep takes up the remaining 50%	Alternation of NREM sleep and REM sleep constitutes the sleep cycle. Each sleep cycle has a duration of approximately 90 min. There are usually 4–5 sleep cycles per night

NREM, non-rapid eye movement; REM, rapid eye movement.

**Box 1:**
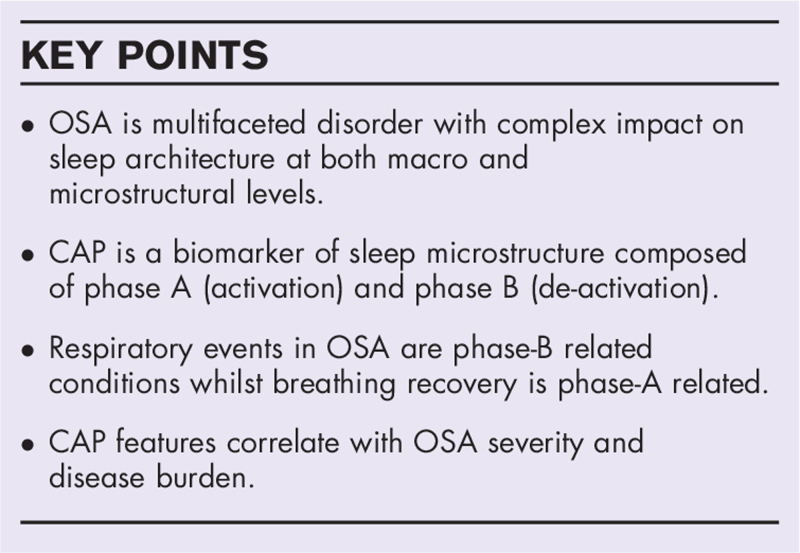
no caption available

## FROM SLEEP MACROSTRUCTURE TO CYCLIC ALTERNATING PATTERN

Sleep architecture is a scaffolding of stages and cycles, an ordered sequence of rooms and stairs functional to the construction of a consistent structure. As in any house or apartment, it is necessary to provide openings in the external wall of the building to give light and air inside, allowing interaction and integration between the external world and the internal habitat. Arousals are not simply short intrusions of wakefulness but represent the domestic windows, equipped with glass and shutters, which indicate the resistance and porosity of the sleep structure to perturbations. A single arousal tends to have limited consequences on sleep continuity and on the individual's health. However, in the texture of physiological sleep, arousals rarely appear as isolated phenomena, while they are often aggregated with other arousals in swarms. Ordered and periodic, sequences of arousals compose the cyclic alternating pattern (CAP) [2]. Details and rules for CAP scoring are outlined in Table 2.

**Table 2:** Main features of sleep microstructure

The rules of cyclic alternating pattern
In all stages of NREM sleep, CAP is organized in sequences
A CAP sequence is made up of a succession of CAP cycles
Each CAP cycle consists of a phase A (activation) and the following phase B (interval)
At least two consecutive CAP cycles are required to define a CAP sequence
Absence of CAP for > 60 s is defined as non-CAP
An isolated phase A, i.e., preceded or followed by another phase A but separated by more than 60 s, is classified as non-CAP
CAP coincides with a condition of physiological instability
Non-CAP translates a condition of physiological stability
Non-CAP and CAP sequences constitute the microstructure of sleep

CAP, cyclic alternating pattern; NREM, non-rapid eye movement.

Based on a 1-min ‘rhythmicity’, during CAP arousability swings in tune with multiple body systems [heart rate (HR), blood pressure, respiration, cerebral blood flow dynamics] [3]. In particular, autonomic functions rise during phase A (arousal) and drop during phase B (interval). Similarly, muscle tone is enhanced during phase A and subsides during phase B. In normal conditions, the 1-min rhythm of CAP is not detectable in REM sleep: this is probably one of the reasons why this sleep stage is dominated by a disordered electroencephalogram (EEG) and vegetative regime. In the more regular non-rapid eye movement (NREM) sleep, the overall percentage of CAP time quantifies the microstrutural parameter called CAP rate.

## FROM PHYSIOLOGY TO PATHOLOGY

**FIGURE 2:**
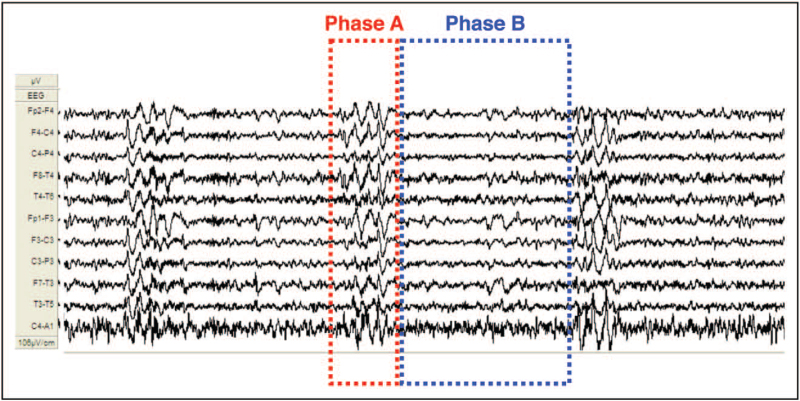
Example of a cyclic alternating pattern cycle: each cyclic alternating pattern cycle is composed of a phase A (activation) and a following phase B (deactivation), outlined by red and blue dotted boxes, respectively. Phase A stands out from the background rhythm and may be represented by variable EEG patterns including delta bursts, vertex transients, polyphasic bursts, alpha rhythm, EEG arousal). Phase B is composed of the background rhythm that separates consecutive A phases. Both phase A and phase B can range between 2 and 60 s. EEG, electroencephalographic.

During university studies of medicine the biochemical and physiology lessons always precede the pathological and clinical exams. In other words, we cannot understand diabetes if we ignore the mechanisms of blood sugar regulation. Accordingly, if sleep architecture is made up of REM and NREM stages, we expect to identify REM- and NREM-related disorders. Moving further, the occurrence of periodic activation (CAP phase A) separated by regular intervals of phasic deactivation (CAP phase B) overcomes the short-sided perspective of isolated arousals and opens the door to pathologies characterized by a typical although exaggerated alternating pattern (Fig. 2). A classical biphasic disease is expressed by obstructive sleep apnea (OSA), which is commonly accompanied by high values of CAP rate. Someone could raise the objection that the increased amounts of CAP in OSA are simply the by-product of mechanical reactions and phasic desaturations, which oblige the sleeping brain to wake up for a few seconds to avoid asphyxia. However, these considerations can be easily dismantled: (1) CAP is a physiological component of NREM sleep occurring in healthy sleepers [4] showing specific features across the lifespan [5]; (2) there are sleep pathologies without respiratory events, that is, insomnia [6], periodic myoclonus [7], NREM parasomnias [8], sleep-related hypermotor epilepsy [9], circadian misalignment [10], mental illness [11] characterized by increased amounts of CAP rate indicating that CAP is not a respiratory-driven artifact, but an intrinsic biological rhythm sensitive to perturbation. In the pathophysiology of OSA, when breathing interruption becomes a menacing factor, the sleeping brain exploits and enhances the biphasic structure of CAP. The deactivation of phase B creates the permissive background of partial or complete airflow blockage, while the readiness of phase A [12] restores respiration and activates for a few seconds both brain and body functions. In the OSA framework, the B phase of CAP is not a trivial interval between consecutive arousals. On the one side, it is the consistent response to the intensity, duration, autonomic tone and motor bursts of the previous A phase [13]. On the other side, the phase B of CAP provides the triggering factor (apnea, hypopnea, flow limitation) of the following arousal response in a restless oscillating chase. Whether phase A or phase B comes first remains an open and probably irrelevant question. What really matters is the fall of the dominant paradigm that EEG arousals are isolated floating rafts. This is the way sleep experts continue to score and interpret arousals. This is why CAP becomes a necessary integration to conventional sleep analysis.

## SHADES OF CYCLIC ALTERNATING PATTERN: THE PHASE A SUBTYPES

**FIGURE 3:**
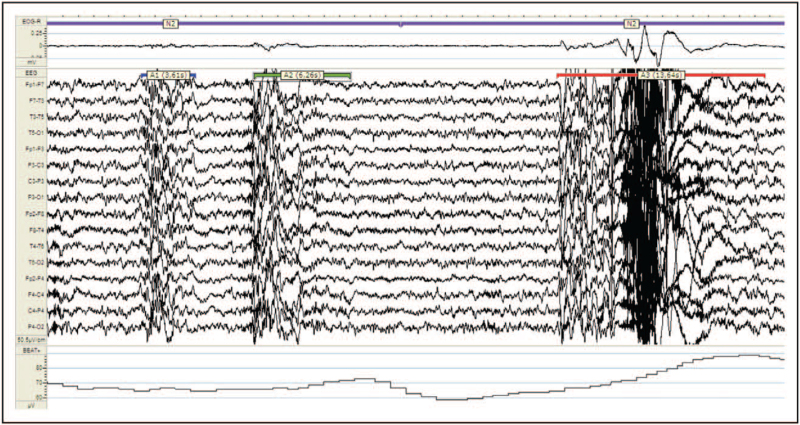
Examples of cyclic alternating pattern subtypes, labeled by colored lines. Note the increasing autonomic impact (heart rate) shifting from subtypes A1, A2, and A3.

The CAP framework not only places standard arousals in the bed of sleep microstructure, but also extends the concept of autonomic activation to EEG features, which are commonly excluded from the scoring criteria. The spontaneous or experimentally-induced increase in respiratory rate and tachycardia that accompany K-complexes and delta bursts indicate that these represent instabilities in the sleep state. The inclusion of slow EEG features in the scoring rules of CAP allows a wider classification of A phases ranging from subtypes A1 (dominated by low-frequency high-voltage waves) to subtypes A3 (with a predominance of high-frequency low-voltage rhythms) with subtypes A2 composed of a balanced mixture of slow and fast EEG oscillations (Fig. 3). Because subtypes A2 and A3 overlap with the amount of EEG arousals in NREM sleep [14], CAP not only includes the counting of standardized features, but permits quantification of subcortical arousals [15]. During slow wave sleep in mice, arousals disclose an ordered chain of events [16^▪▪^]. In detail, an increase of cortico–hippocampal coherence precedes the increase of this parameter in cortico–cortical networks indicating that neural processes in sub-cortical structures (such as the hippocampus) provide a preparation for the emergence of an EEG arousal. Loss of entropy starts approximately 4 s before the arousal onset, and coincides in time with a progressive increase in cortico-hippocampal frequency coherence. In rodents, two distinct arousal-related pathways can be revealed, both arising from the subcortical pontine parabrachial nucleus (PBN). Although mild stressors reach the PBN leading to empowerment of high-voltage synchronized slow wave sleep, major distress activates the lateral subregions of this area evoking a powerful basal forebrain arousal and promoting the appearance of faster rhythms [17]. Similarities between the arousal-related electrical reactions in rodents and CAP subtypes (A1 reflecting mild stress, A2 and A3 triggered by strong stress) have been highlighted in a recent study conducted on OSA patients [18^▪^]. Patients with severe OSA suffer from altered homeostatic balance, reflected through the marked reduction of subtypes A1, K-complexes and N3, while the ultradian process expressed by subtypes A2 and A3 is preserved [19].

## CYCLIC ALTERNATING PATTERN AND OBSTRUCTIVE SLEEP APNEA: THE HYPERSOMNOLENCE MAZE

According to Punjabi (2021) [20], *to focus only on arousals is akin to only seeing the tip of the iceberg and neglecting the complexity of events that characterize sleep continuity. Because sleep fragmentation is commonly operationalized using arousals, the omission of other, and perhaps more sensitive, measures of sleep state instability could certainly explain the poor association between arousal frequency and daytime sleepiness in sleep apnea*. Hypersomnolence is certainly a puzzling issue modulated by manifold factors including synaptic messengers (e.g., orexin) and homeostatic balances (e.g., sleep deprivation). Excessive daytime sleepiness (EDS) is a common but not mandatory finding in OSA patients. A recent study showed that the ESS (Epworth Sleepiness Scale) is not correlated with sleep disordered breathing (SDB) at mild to moderate levels in women and has a smaller association than in men with severe SDB [21]. Complaints of tiredness and daytime sleepiness, ESS and MSLT (multiple sleep latency test) scores are similar in patients with OSA (apnea hypopnea index [AHI] > 5) and upper airway resistance syndrome (AHI < 5) indicating that daytime sleepiness is not reflected by the amount of respiratory events or by EEG arousals [22]. Once again the magnifying glass of CAP can shed light on these controversial findings. In a retrospective study [23], 38 male patients with moderate to severe OSA were divided into two subgroups, excessive sleepiness or nonexcessive sleepiness, according to the score of the ESS. There was no difference between the two subgroups in clinical characteristics and macrostructural parameters of sleep. Although the amount of arousals is often used to quantify the level of sleep fragmentation and high-arousal values can be associated with daytime sleepiness [24], the number of arousals and arousal index did not differ significantly in the two subgroups. In contrast, CAP metrics accompanied consistently the complaint of vigilance impairment. In detail, the subgroup with excessive sleepiness showed higher amounts of CAP time, CAP rate (especially subtype A2), number of CAP cycles and a lower mean duration of phase B.

**FIGURE 4:**
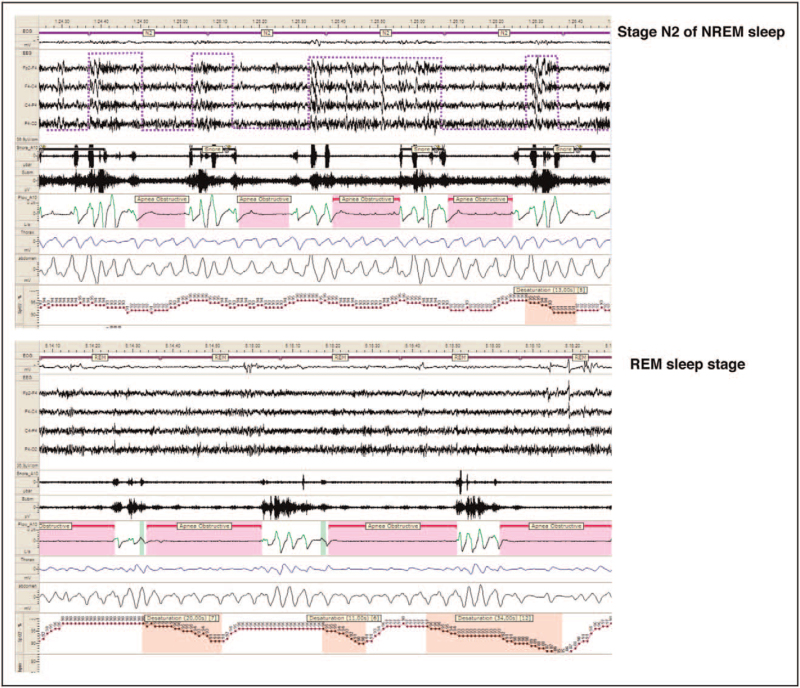
Polysomnography of an adult male patient with untreated severe obstructive sleep apnea during NREM sleep (stage N2) and REM sleep. Note the synchronized oscillations of sleep microstructure (cyclic alternating pattern subtypes A3) with obstructive apneas (during phase B of cyclic alternating pattern), the regular duration of intermittent desaturation and transient increase of heart rate in NREM sleep. Conversely, apneas are longer in REM sleep, where the ‘modulatory’ effect of cyclic alternating pattern is lacking. NREM, non-rapid eye movement; REM, rapid eye movement.

CAP features also portray the severity of OSA as subtypes A1 usually prevail in milder phenotypes, while subtypes A2 and A3 dominate among patients with moderate-to-severe OSA [18^▪^]. In the latter condition, the persistence of mechanical and functional triggers (hypoxia, hypercapnia, autonomic changes and respiratory effort) progressively pushes the CAP system from a sleep-protecting role, that is, CAP subtypes A1 towards an arousal-promoting reaction, that is, CAP subtypes A2 and A3, leading to sleep fragmentation [8]. Increased amounts of CAP rate correlated with fatigue and sleepiness in adults are typical features of the upper airway resistance syndrome [25]. To further complicate the scenario, recent evidence indicates that REM-OSA patients are affected by more severe EDS compared with NREM-OSA, despite the lower total AHI values [26^▪^]. Previous investigation ascertained that the absence of the Janus-faced CAP fluctuations during REM sleep and the physiological muscle atonia of this sleep condition expose patients to longer and more severe apnea events [27] (Fig. 4). The random distribution of arousals in REM sleep is consistent with the well-known irregularity of vegetative functions, in contrast to the more controlled autonomic activities in NREM sleep where their waxing and waning behavior coincides with the periodicity of CAP. In sleep apnea, the A phases interrupt at regular intervals the phase-B related respiratory event during NREM sleep, the so called CAP-dominant OSA; in contrast, the lack of a ‘protective’ cyclic activity in REM sleep favors the development of irregular apneas and hypopneas [28]. According to Mazzotti *et al.*[29^▪▪^], excessively sleepy OSA patients from the Sleep Heart Health Study are at higher risk for incident cardiovascular disease, coronary heart disease and heart failure. Sleepy OSA patients also present autonomic balance impairment. In particular, lower baroreflex sensitivity and abnormal nocturnal HR variability exert a relevant impact during the different stages of nocturnal sleep, but major effects occur during N3 [30]. Notably, an increase of the low-frequency band, increase of the low-frequency/high-frequency ratio and decrease in the high-frequency band have been described during CAP in stages N2 and N3 [31]. In conclusion, EDS in OSA is a multifactorial maze and probably relies on manifold polysomnography and clinical parameters. Furthermore, in persistently sleepy OSA patients, despite their adherence to therapy, alternative patholgies need to be ruled out including depression, insomnia, restless legs syndrome, narcolepsy, hypothyroidism, circadian sleep disorders [32^▪▪^].

## CYCLIC ALTERNATING PATTERN AND CONTINUOUS POSITIVE AIRWAY PRESSURE: THE RESILIENCE OF THE SLEEPING BRAIN

**FIGURE 5:**
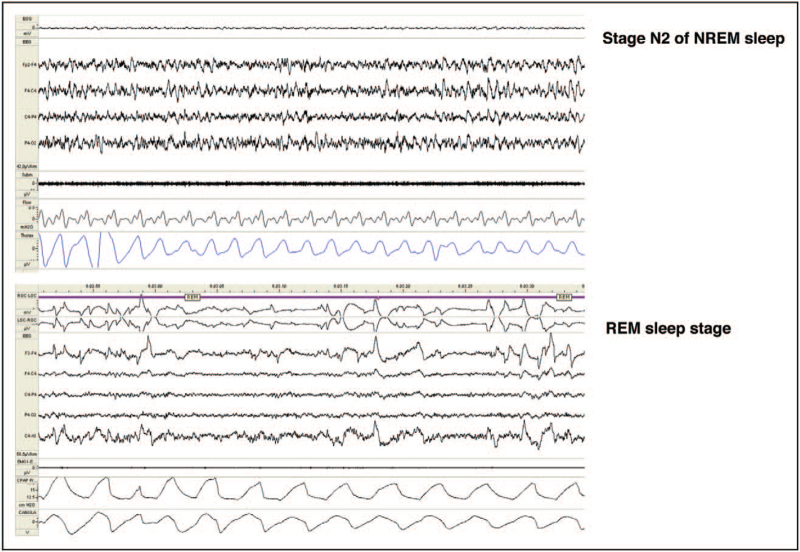
Same patient of Fig. 4 after 30-days of continuous positive airway pressure therapy.

Previous investigation has ascertained that CPAP (continuous positive airway pressure) at efficacious pressure improves daytime somnolence in OSA patients only after a few nights of therapy [33] (Fig. 5). CPAP leads to an immediate rebound of N3 and REM sleep after the first night of treatment [34]. In a study carried out in OSA patients after a single night of CPAP treatment, a significant correlation was found between the reduction of CAP rate and the scores of subjective sleepiness. In contrast, no significant relationship emerged between the number of arousals and diurnal vigilance even when objective tests (MSLT) were accomplished before and after ventilatory treatment [35]. Recovery of normal sleep parameters occur at different time intervals. Under regular and effective ventilatory treatment, phases A2 and A3 of CAP recover normal values during night 1, CAP rate during night 2, N3 during night 3, while stages N1 and N2 return to normal only after 30 days. Overall, microstructural parameters recover normal values more rapidly than conventional measures. However, CAP subtypes A1 deficiency caused by severe OSA is still identifiable after prolonged CPAP utilization and is commonly associated with subtle cognitive impairment [19].

## CONCLUSION

In normal individuals, arousals occur more frequently during CAP (40 events per hour) than in total sleep time (18 events per hour), NREM sleep (20 events per hour), and REM sleep (12 events per hour) [35]. If conventional ‘arousals’ coincide with subtypes A2 or A3, the CAP rules allow a more inclusive evaluation of sleep instability made up also of transient slow activation complexes (K-complexes, delta burst, subtypes A1). Therefore, confining sleep consolidation to conventional arousals neglects the role of cortical and subcortical phenomena, which are pieces of a complex structural pattern. The regular phase-B intervals favors perpetuations of cyclic events during sleep, as occurs in OSA.

Involved in the pathophysiology of OSA, CAP can shed light on the severity of SDB and can be used to predict disease burden. Although EDS in OSA is a complex issue, sleep microstructure covers part of the hypersomnolence puzzle, with higher amounts of CAP in sleepy OSA patients. Finally both conventional and microstructural sleep metrics undergo gradual but independent changes during prolonged CPAP utilization. The bidirectional and simultaneous relationship between ‘upper floors’ (brain instability) and ‘lower floors’ (cardiopulmonary and autonomic systems) needs to be considered in OSA to properly understand the pathophysiological mechanisms of this biphasic sleep disorder.

## Acknowledgements

*The article is dedicated to Mario Giovanni Terzano, father of CAP (cyclic alternating pattern). The authors wish to acknowledge Margherita Soglia, Silvia Pizzarotti, Anna Abramo, Francesca Alessandrini and all the team members of the Sleep Disorders Center, Parma, for their irreplaceable support.*

### Financial support and sponsorship

*None.*

### Conflicts of interest

*There are no conflicts of interest.*

## REFERENCES AND RECOMMENDED READING

Papers of particular interest, published within the annual period of review, have been highlighted as:

▪ of special interest
▪▪ of outstanding interest
